# Proteomic analyses reveal cystatin c is a promising biomarker for evaluation of systemic lupus erythematosus

**DOI:** 10.1186/s12014-023-09434-9

**Published:** 2023-10-18

**Authors:** He Huang, Yukun Zhang, Lan Gui, Li Zhang, Minglong Cai, Yujun Sheng

**Affiliations:** 1 Department of Dermatology, The First Affiliated Hospital of Anhui Medical University, Hefei, Anhui China; 2 Department of Rheumatology and Immunology, The First Affiliated Hospital of USTC, Division of Life Sciences and Medicine, University of Science and Technology of China, Hefei, Anhui China; 3 Department of Dermatology, China-Japan Friendship Hospital, Beijing, China

**Keywords:** Systemic lupus erythematosus, Proteomics, Cystatin C, Neutrophil activation, Biomarker

## Abstract

**Background:**

Systemic lupus erythematosus (SLE) is an autoimmune disease with multiple organ involvement, especially the kidneys. However, the underlying mechanism remains unclear, and accurate biomarkers are still lacking. This study aimed to identify biomarkers to assess organ damage and disease activity in patients with SLE using quantitative proteomics.

**Methods:**

Proteomic analysis was performed using mass spectrometry in 15 patients with SLE and 15 age-matched healthy controls. Proteomic profiles were compared in four main subtypes: SLE with proteinuria (SLE-PN), SLE without proteinuria (SLE-non-PN), SLE with anti-dsDNA positivity (SLE-DP), and SLE with anti-dsDNA negativity (SLE-non-DP). Gene ontology biological process analysis revealed differentially expressed protein networks. Cystatin C (CysC) levels were measured in 200 patients with SLE using an immunoturbidimetric assay. Clinical and laboratory data were collected to assess their correlation with serum CysC levels.

**Results:**

Proteomic analysis showed that upregulated proteins in both the SLE-PN and SLE-DP groups were mainly mapped to neutrophil activation networks. Moreover, CysC from neutrophil activation networks was upregulated in both the SLE-PN and SLE-DP groups. The associations of serum CysC level with proteinuria, anti-dsDNA positivity, lower complement C3 levels, and SLE disease activity index score in patients with SLE were further validated in a large independent cohort.

**Conclusions:**

Neutrophil activation is more prominent in SLE with proteinuria and anti-dsDNA positivity, and CysC is a promising marker for monitoring organ damage and disease activity in SLE.

**Supplementary Information:**

The online version contains supplementary material available at 10.1186/s12014-023-09434-9.

## Introduction

Systemic lupus erythematosus (SLE) is mediated by autoreactive autoantibodies that damage multiple tissues and organs [1]. At least 50% of patients with SLE develop lupus nephritis (LN), which is a serious manifestation of SLE [2]. However, the pathogenesis of SLE remains unclear.

Proteinuria, creatinine ratio, creatinine clearance, and anti-dsDNA antibodies are common markers for the detection and assessment of LN [3]. Autoantibodies, including antinuclear antibodies (ANAs), anti-dsDNA antibodies, anti-histone antibodies, anti-SSA/Ro and anti-SSB/La antibodies, and anti-phospholipid antibodies, are involved in multiple organ damage, especially the kidneys [1]. Moreover, anti-DNA antibody levels in the serum of patients with SLE fluctuate with disease activity [4]. Anti-dsDNA antibody is the most widely used index in laboratory tests and is part of the classification criteria for SLE. They are present in nearly 80% of patients with LN and can cross-react with glomerular antigens [5]. A study showed that anti-dsDNA levels were correlated with disease severity in 40 patients with untreated LN [6]. In murine models of SLE, organ damage can be prevented or even reversed by blocking the production of pathogenic anti-dsDNA antibodies [4]. Anti-dsDNA antibodies were explored as potential therapeutic targets for SLE [7–9]. Proteinuria is one of the most common manifestations of kidney involvement in SLE. Currently, it is recommended that patients with overt proteinuria (e.g., urine protein: creatinine ratio > 500 mg/g) undergo kidney biopsy [10]. Proteomics-based studies have been applied in SLE to discover differentially regulated proteins [11, 12]. In some reports, C4d, serum HMGB1, and several cytokines proposed as potential biomarkers for screening SLE [13–15]. Although these potential biomarkers were mainly expressed in SLE, it is important to identify sensitive and specific biomarkers of renal disease activity.

In the present study, whole proteomics of patients with SLE and healthy controls was performed using mass spectrometry to identify and establish biomarkers to achieve a more accurate and reliable evaluation of renal damage and disease activity in patients with SLE.

## Materials and methods

### Sample collection and preparation

This study was approved by the scientific ethics committee of The First Affiliated Hospital of Anhui Medical University. Written informed consent was obtained from all participants prior to the study. Among these, 15 patients with SLE and 15 age- and sex-matched healthy controls (HCs) were used for proteomics; another 200 patients in the SLE cohort were used for serum biomarker assessment. Clinical information was abstracted from the medical records. Disease activity was recorded using the SLE disease activity index (SLEDAI) score. Patients with SLE were classified into two comparison groups: SLE patients with proteinuria (SLE-PN) versus SLE patients without proteinuria (SLE-non-PN) and SLE patients with anti-dsDNA positivity (SLE-DP) versus SLE patients with anti-dsDNA negativity (SLE-non-DP).

### Preparation of peripheral blood mononuclear cells (PBMCs) and protein extraction

Blood was collected into 10-mL ethylenediaminetetraacetic acid evacuated tubes (BD, USA) for PBMC isolation. PBMCs were isolated with density-gradient centrifugation in Ficoll Paque Premium (GE Healthcare, USA) and washed twice in phosphate buffered saline. The PBMC samples were stored at − 80°C prior to use. Protein was extracted from isolated PBMCs on ice using a high-intensity ultrasonic processor (Scientz) in lysis buffer (8 M urea, 1% protease inhibitor cocktail). The remaining debris was removed by centrifugation at 12,000×*g* and 4 °C for 10 min. Finally, the supernatant was collected, and the protein concentration was determined using a BCA kit according to the manufacturer’s instructions.

### Measurement of cystatin C (CysC) in serum samples

We collected 5 mL of peripheral blood from each individual for serum biomarker assessment. Serum was separated by centrifugation at 3000 rpm for 15 min within 2 h after sample collection. To detect serum CysC levels, enzyme-linked immunosorbent assay was performed by Jiuqiang Biotechnology (Beijing, China) in accordance with the manufacturer’s instructions.

### Mass spectrometry and data analyses

In brief, the peptide was dissolved by liquid chromatography with mobile phase A and superheated by Nano Elute High performance liquid phase system for separation. Mobile phase A is an aqueous solution containing 0.1% formic acid and 2% acetonitrile. mobile phase B is a solution containing 0.1% formic acid and 100% acetonitrile. liquid phase gradient setting: 0–70 min, 4%–22%B; 70–84 min, 22% to 30%B; 84–87 min, 30%–80%B; 87–90 min, 80%B, flow rate maintained at 450.00 nL/min. The peptides are separated by an ultra-high performance liquid phase system and injected into the Capillary ion source for ionization and then analyzed by TIMES TOF Pro mass spectrometry. The ion source voltage was set to 2.0 kV, and the parent ion of the peptide segment and its secondary fragments were detected and analyzed using high-resolution TOF. The secondary mass spectrometry scan range is set to 100–1700. The data acquisition mode uses parallel cumulative serial fragmentation (PASEF) mode. A secondary spectrum with charge number of parent ions in the range of 0–5 was collected by PASEF mode for 10 times after primary mass spectrometry collection. The dynamic exclusion time of series mass spectrometry scanning was set to 30 s seconds to avoid repeated scanning of parent ions. Detailed analyses were described as previously [16]. For quality control of the expression data, we filtered low-abundance proteins (< 1 in > 80% of samples) and converted the expression data into a logarithmic form, which met the normal distribution. The Limma package (v.3.50.1) [17] was applied to define differentially expressed proteins (DEPs) between the two groups with a 1.5-fold change and P-value < 0.05. Cluster Profiler is an R package used to compare biological themes among gene clusters [18].

### Statistical analyses

All statistical analyses were performed using SPSS 22.0 (IBM, Armonk, NY, USA). Continuous variables are presented as mean ± standard deviation if normally distributed (according to the Shapiro–Wilk test) and analyzed using the independent t-test; otherwise, they were presented as median (range) and analyzed using the Mann–Whitney U-test. Categorical variables are presented as n (%) and were analyzed using the chi-square test or Fisher’s exact test. Two-sided P-values < 0.05 were considered statistically significant. Student’s t-tests were used for comparisons between two groups, and one-way analysis of variance was used for multi-group comparisons using R (v.3.6.1).

## Results

### Patient characteristics

Patients with SLE and age- and sex-matched HCs were used for proteomics, and clinical information, including sex, age, autoantibodies, and proteinuria, was collected (Table 1). To assess the correlation between CysC and clinical and laboratory data, another cohort including 200 patients with SLE was included (Table 2). The average CysC level was 1.55 mg/L, and the average SLEDAI score was 12.55 points.

**Table 1:** Characteristics of patients with SLE for proteomics

Characteristic	HC	SLE
n	15	15
Age, year (mean ± SD)	33.53 ± 6.47	34.53 ± 8.15
Age onset, year (mean ± SD)	–	29.73 ± 7.79
SLEDAI (mean ± SD)	–	6.4 ± 4.52
Proteinuria (> 0.5 g/24 h), n, %	–	6, 67%
Malar erythema, n, %	–	8, 53.33%
Phontaesthesia, n, %	–	4, 26.67%
Mucosal ulcers, n, %	–	1, 6.67%
Arthritis, n, %	–	2, 13.33%
Pleurisy, n, %	–	0, 0%
Psychosis, n, %	–	2, 4.44%
Fever, n, %	–	0, 0%
Lupus encephalopathy, n, %	–	4, 26.67%
Alopecia	–	2, 13.33%
ANA, n, %	–	15, 100%
Anti-JO-1 antibody, n, %	–	1, 6.67%%
Anti-SM antibody, n, %	–	5, 27.78%
Anti-SSA antibody, n, %	–	10, 55.56%
Anti-SSB antibody, n, %	–	3, 16.67%
Low C3, n, %	–	12, 80%
Anti-dsDNA antibody, n, %	–	6, 40%

**Table 2:** Characteristics of patients with SLE for serum biomarker assessment

Characteristic	SLE
N	200
Female, n, %	182, 91%
Age, year (mean ± SD)	38.06 ± 12.78
SLEDAI (mean ± SD)	12.55 ± 6.97
CysC (mean ± SD)	1.55 ± 0.94
Proteinuria (> 0.5 g/24 h), n, %	63, 31.5%
Hematuria, n, %	76, 38%
Cylindruria, n, %	11, 5.5%
Malar erythema, n, %	5, 66.67%
Phontaesthesia, n, %	2, 28.57%
Mucosal ulcers, n, %	15, 7.5%
Lupus headache, n, %	6, 3%
Arthritis, n, %	94, 47%
Rash, n, %	55, 27.5%
Pleurisy, n, %	1, 0.5%
Pyuria, n, %	69, 34.5%
Psychosis, n, %	5, 2.5%
Fever, n, %	72, 36%
Lupus encephalopathy, n, %	1, 0.5%
Alopecia, n, %	21, 10.5%
Low C3, n, %	144, 72%
Anti-dsDNA antibody, n, %	115, 57.5%

### Neutrophil activation network associated with organ damage and disease activity in SLE

**Fig. 1:**
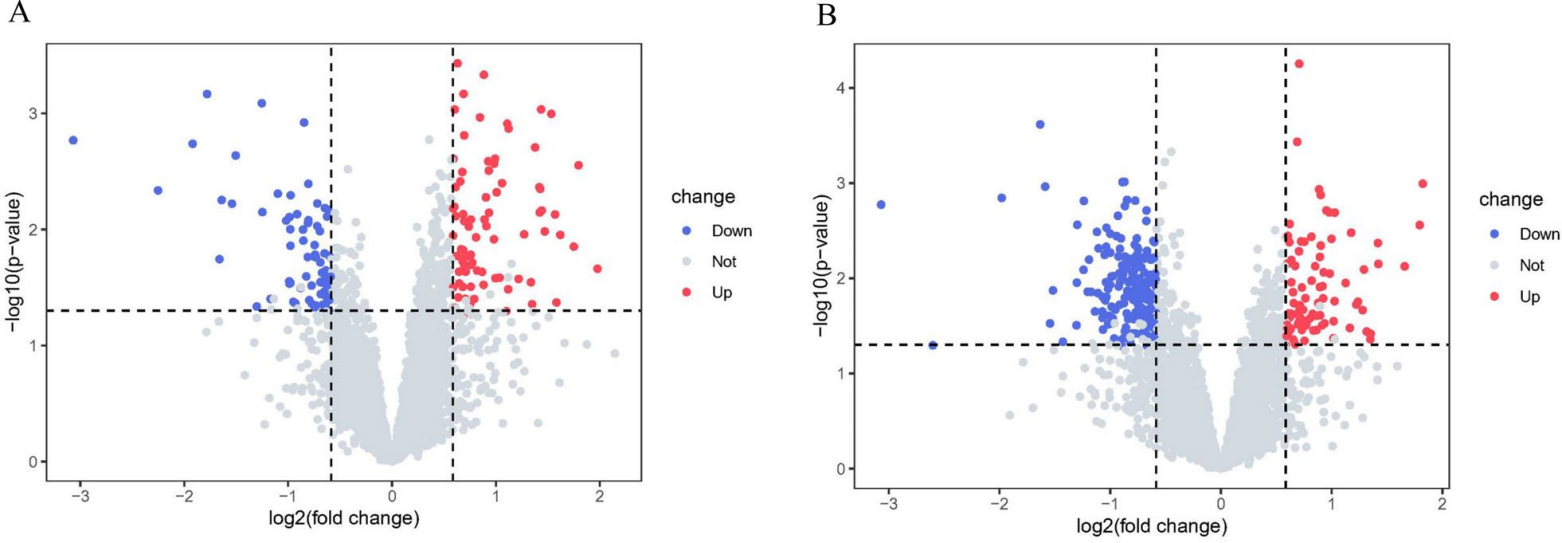
Protein Expression Heterogeneity in Different Subgroups of Systemic Lupus Erythematosus (SLE). Volcano plot showing differential expression proteins (DEPs) in **A** SLE with proteinuria (SLE-PN) versus SLE without proteinuria (SLE-non-PN) and **B** SLE with anti-dsDNA positivity (SLE-DP) versus SLE without anti-dsDNA positivity (SLE-non-DP) groups. DEPs were defined as fold change > 1.5 and p < 0.05, red dots represent up-regulated proteins and  blue dots represents down-regulated proteins

**Fig. 2:**
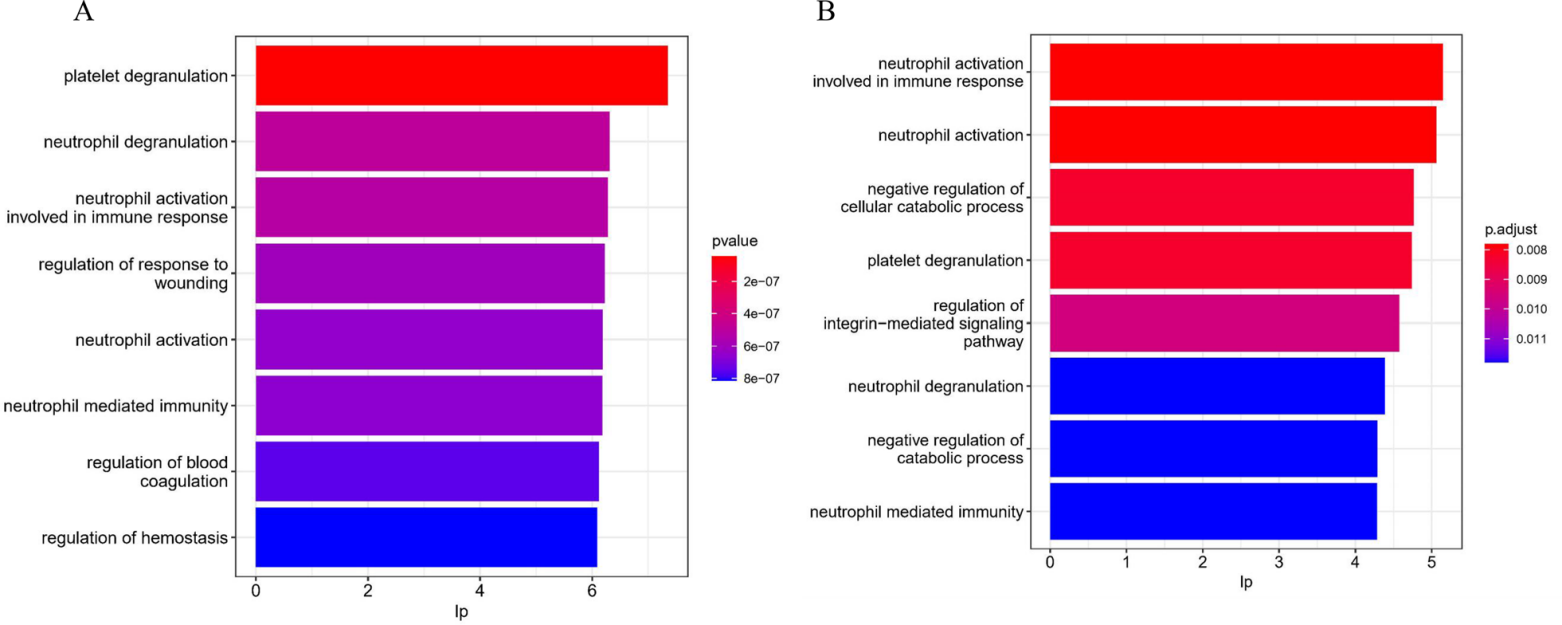
Results of GO enrichment analysis of DEPs in neutrophil activation networks in **A** SLE-PN versus SLE-non-PN and **B** SLE-DP versus SLE-non-DP groups

**Fig. 3:**
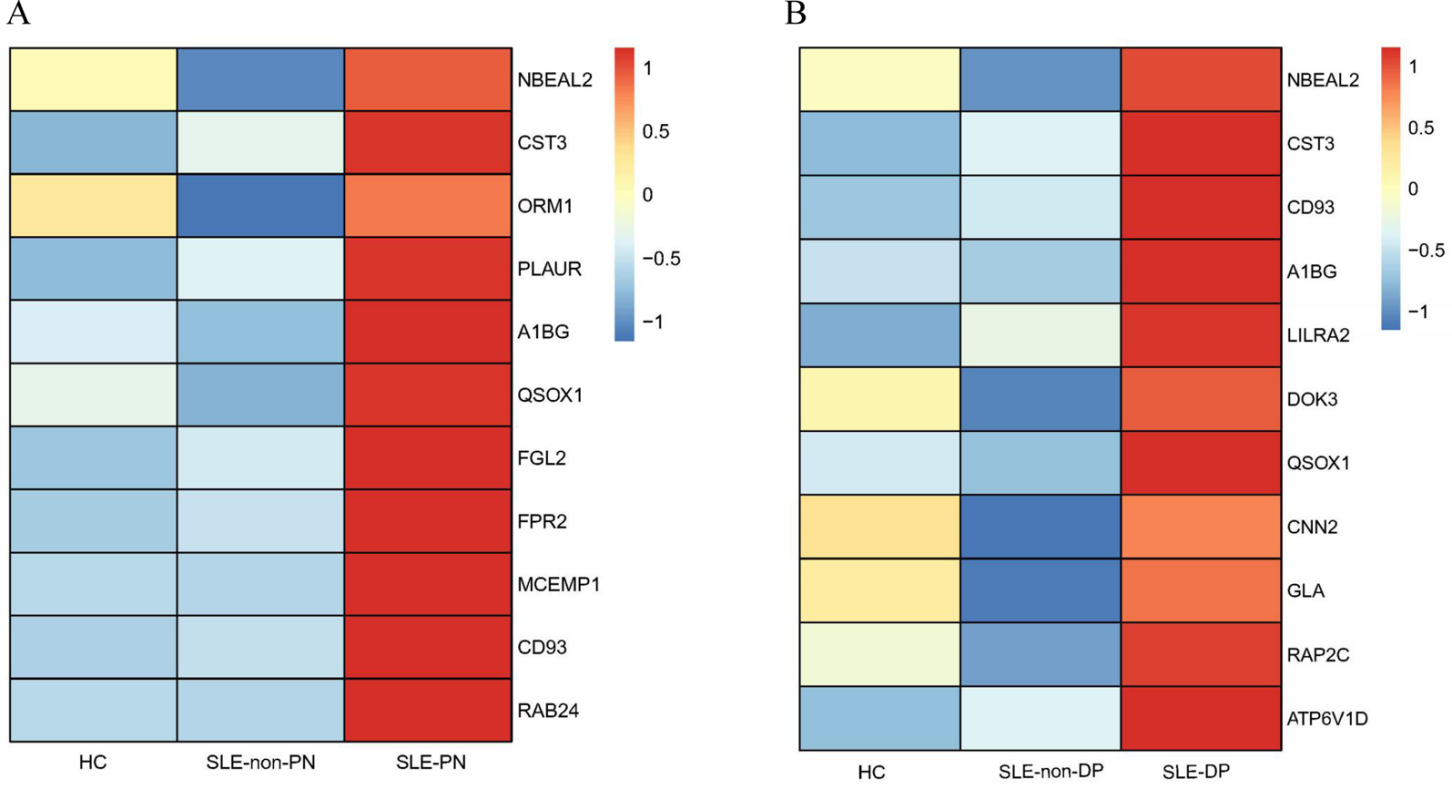
Heatmap showing the expression of core proteins that contribute to neutrophil activation pathway enrichment (red, high abundance; blue, low abundance; protein names are represented by their encoding genes). **A** SLE-PN versus SLE-non-PN groups and HCs and **B** SLE-DP versus SLE-non-DP groups and healthy controls (HCs)

**Fig. 4:**
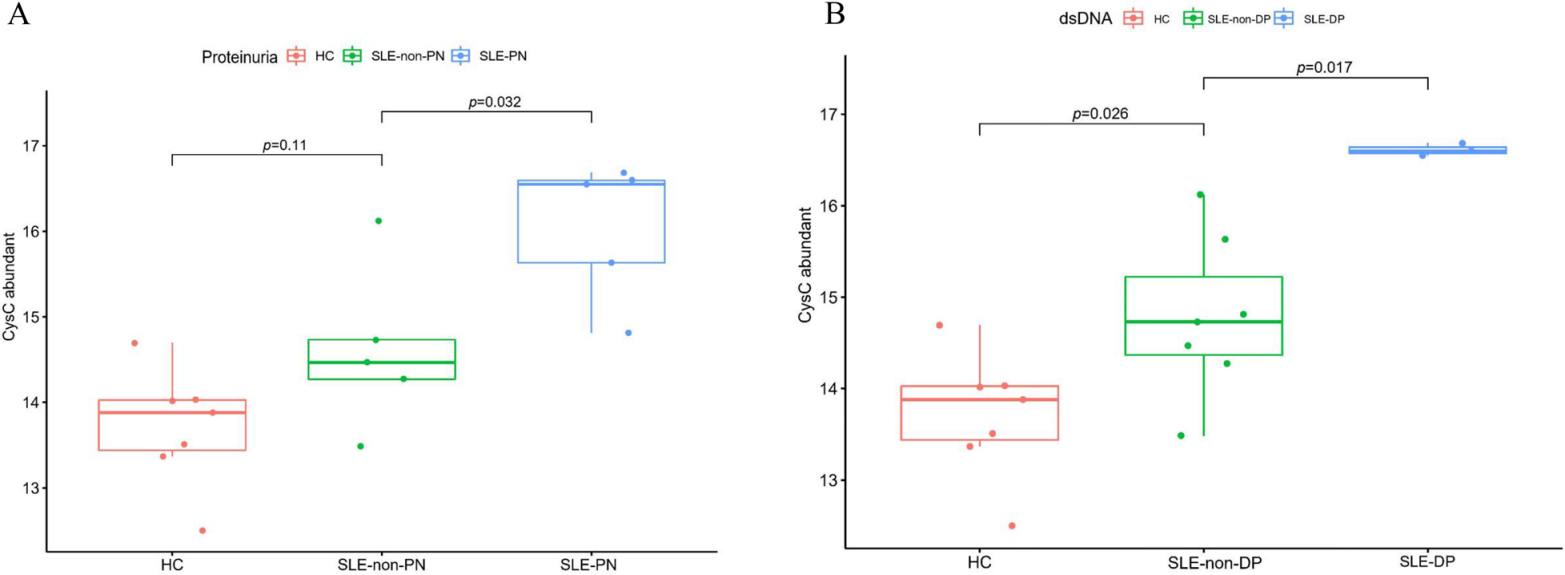
Boxplot showing Cystatin C (CysC) levels in **A** HCs, SLE-non-PN and SLE-PN groups and **B** HCs, SLE-non-DP and SLE-DP groups

Using mass spectrometry, we identified a total of 4830 proteins in the 15 HCs and 15 patients with SLE. To identify protein markers associated with disease subtypes, we performed differential protein analysis across two comparison groups: SLE-PN versus SLE-non-PN and SLE-DP versus SLE-non-DP. Indeed, 80 proteins were more abundant and 74 were less abundant at the proteomic level in the SLE-PN group than in the SLE-non-PN group, whereas 87 proteins were more abundant and 216 were less abundant in the SLE-DP group than in the SLE-non-DP group (fold change > 1.5 or < 0.67; P < 0.05; Fig. 1, Additional file 1: Data S1). Furthermore, we performed gene ontology biological process analysis of DEPs from the SLE-PN and SLE-DP groups and found that upregulated proteins in both comparison groups were mainly mapped to neutrophil activation networks (Fig. 2), including CysC, FGL2, and CD93 (Fig. 3). Previous studies reported that CysC serves as a biomarker of kidney function [19]. Here, our proteomic analysis showed that CysC was upregulated in the SLE-PN and SLE-DP groups (Fig. 4), suggesting that CysC is a biomarker of organ damage and disease activity in SLE.

### CysC promote kidney damage and disease activity in SLE

**Fig. 5:**
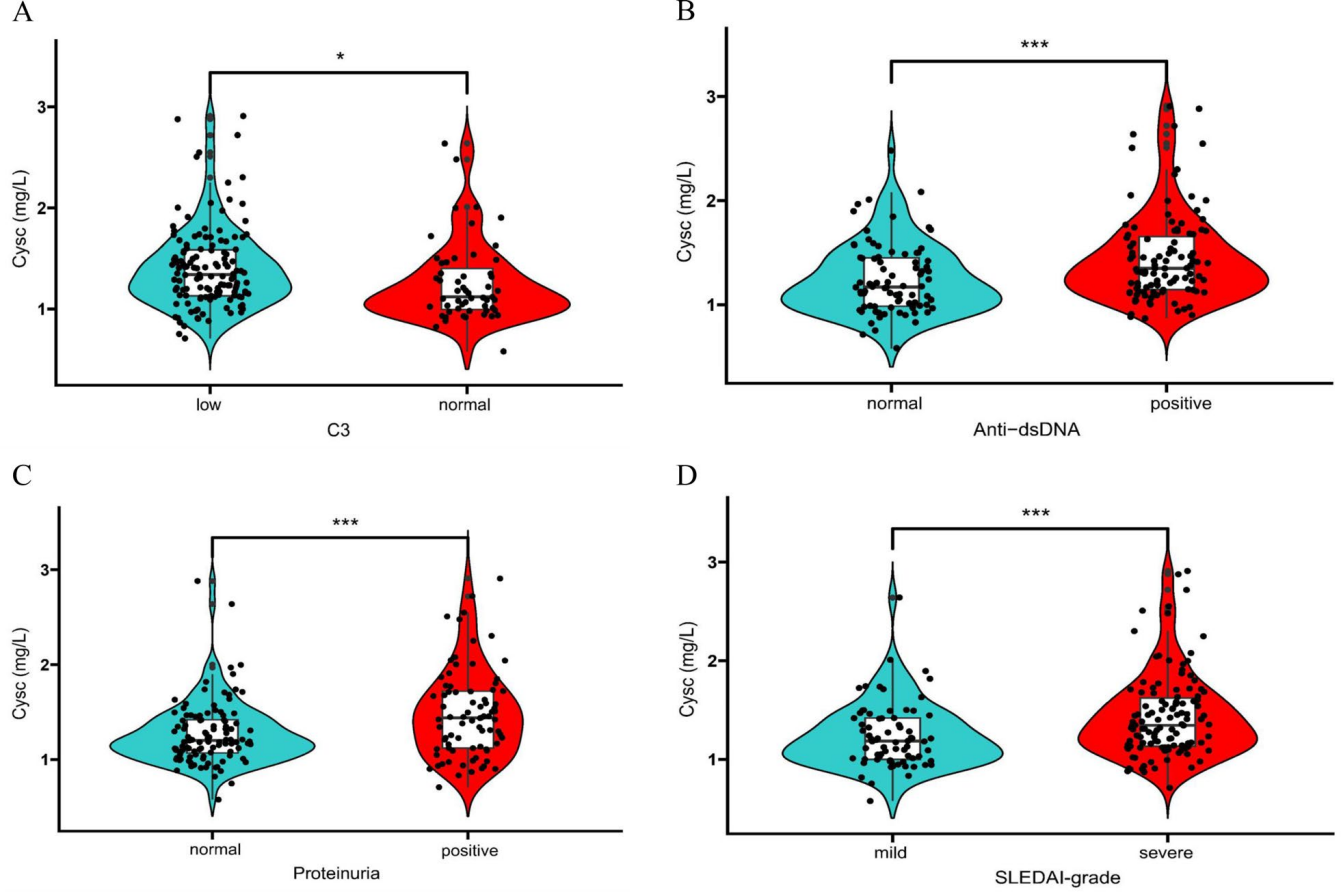
Boxplot showing CysC levels in **A** normal complement C3 versus low complement C3, **B** SLE-non-DP versus SLE-DP groups, and **C** SLE-non-PN versus SLE-PN groups. **D** SLE disease activity index (SLEDAI) score: mild versus severe (a score of 0–8 indicated mild severity, and a score of ≥ 9 indicated moderate-to-severe). *P < 0.05, **P < 0.01, ***P < 0.001

To further understand the role of CysC in SLE, CysC levels were measured in 200 patients with SLE. We then evaluated the association between CysC and clinical and laboratory data of patients with SLE. Disease activity in patients with SLE was assessed using SLEDAI; a SLEDAI score of 0–9 indicated mild severity, and a SLEDAI score of > 9 indicated moderate-to-severe SLE. Logistic regression analysis was used to examine whether CysC was associated with proteinuria, hematuria, anemia, neutropenia, lower lymphopenia, complement 3 (C3) levels, anti-dsDNA, and SLEDAI scores, which revealed significant associations between CysC levels and proteinuria, anti-dsDNA, lower C3 levels, and SLEDAI scores in patients with SLE (Fig. 5A–D). We also performed a receiver operating characteristic (ROC) curves analysis to assess the CysC expression is a biomarker for SLEDAI and kidney involvement in SLE, with the areas under the ROC curves of 0.672 and 0.729, respectively (Additional file 2: Figure S1).

## Discussion

The identification of new clinical and laboratory biomarkers is crucial for early detection and supervision of disease activity and renal damage in patients with SLE. Mass spectrometry proteomics can help gain insight into protein abundance from large-scale studies of biological systems [20]. In this study, we performed whole proteomics analysis of patients with SLE and HCs using bioinformatics and biomarker validation, and CysC was evaluated as a biomarker for renal impairment and disease activity in Chinese patients with SLE.

Anti-dsDNA antibody levels have been associated with disease activity and LN [21]. Periodic measurement of anti-dsDNA antibody titers is considered essential once SLE is diagnosed to monitor disease progression [22, 23]. Additionally, accurate evaluation of proteinuria is critical to the clinical management of LN because it is currently the most important biomarker of disease activity and renal prognosis available [24, 25]. Based on these evidence, we classified patients into four subgroups: SLE-PN, SLE-non-PN, SLE-DP, SLE-non-DP and compare their proteomic difference.

There is a higher prevalence of neutrophils in patients with SLE, which are specialist cells of the innate immune system [26]. Neutrophils include normal density neutrophils (NDNs) and low-density granulocytes (LDGs). LDGs induce increased endothelial damage and vascular dysfunction in vitro, through their enhanced ability to synthesize and extrude neutrophil extracellular traps (NETs) [27]. Neutrophil transcripts were enriched in patients with active renal disease [28]. Neutrophil degranulation and activation were upregulated in active renal involvement patients with SLE [29]. In SLE, NETs stimulate the production of proinflammatory cytokines and type I interferons (IFNs), promote immune cell maturation, and contribute to tissue damage [30]. Increased numbers of apoptotic neutrophils have been found in patients with SLE and are related to anti-dsDNA antibody levels [31]. Thus, our finding of upregulated proteins in both comparison groups was mainly mapped to neutrophil activation networks with anti-dsDNA antibody positivity. A previous study also identified neutrophils/LDGs producing NETs in SLE-affected kidneys, which were correlated with anti-dsDNA antibody levels in these patients [32]. Proteins were mainly mapped to neutrophil activation networks, in addition to CysC, there are other proteins including NBEAL2, FGL2, CD93. NBEAL2 (neurobeachin-like 2) encodes a protein of 2754 amino acids and expressed in in platelets, monocytes, and neutrophils [33, 34]. Neutrophils and NK cells function abnormally in the Nbeal2-deficient immune system [35]. NBEAL2 is critically important for neutrophils as regulator of specific granule release [36]. Fibrinogen-like 2 (FGL2) is a member of the fibrinogen superfamily that exists in a membrane-bound and soluble form [37]. FGL2 expressed on neutrophils in addition to endothelial cells, macrophages and regulatory T cells (Treg) [38]. FGL2 mRNA expression is elevated in chronic kidney disease (CKD), and higher FGL2 levels are associated with fibrosis and worse outcomes [39]. Li et al. illustrated that NETs formation was regulated by the FGL2 in liver injury [40]. CD93 is a C-type lectin-like domain (CTLD) containing glycoprotein expressed on endothelial cells, platelets and a variety of leukocytes [41]. CD93 is upregulated on the surface of neutrophils upon activation in vitro, suggesting it is present in neutrophil granules [42]. Moosig et al. showed that CD93 expression was unchanged between SLE patients and controls; however, CD93 expression was inversely correlated with prednisone dosage [43]. These findings explain the association of neutrophil activation networks with organ damage and disease progression in SLE.

CysC is a member of the CysC superfamily that is encoded by the housekeeping gene CST3 and is associated with numerous immunological processes, which results in inflammatory autoimmune diseases and tumor development (multiple myeloma and breast cancer) [44, 45]. A significant association exists between high serum CysC levels, proteinuria, and reduced glomerular filtration rate, which could reflect renal damage and impaired renal function [19]. Multiple studies have shown that CysC levels are higher in patients with SLE than in controls [46–48]. A meta-analysis showed serum CysC is clearly superior to creatinine as a marker of GFR prediction and earlier detection of renal failure [49]. Consistently, our proteomic analysis revealed that CysC levels were upregulated in both the SLE-PN and SLE-DP groups. We also validated the significant associations between CysC level and proteinuria, anti-dsDNA antibody, lower C3 level, and SLEDAI scores in another independent cohort of patients with SLE. Reduced complement C3 and C4 protein levels and high anti-dsDNA antibody levels occur with active disease in SLE [50]. These findings suggest that CysC is a biomarker for kidney damage and disease activity in SLE. Additional routine renal function tests have identified CysC as a better marker of kidney function than creatinine due to its constant secretion and non-dependence on filtrate or other factors, such as muscular mass and sex [19, 51] and SLE is an autoimmune disease that mainly affects female patients [52]. Thus, it is thought to be a noninvasive and reliable measure to estimate kidney function.

Our study has some limitations. First, other proteins in neutrophil activation networks have not been validated in independent samples. Second, the majority of patients with SLE presented with a chronic disease, and some of them were receiving systemic therapy, which may affect CysC levels. Finally, we did not test CysC levels in a matched healthy control group.

## Conclusions

Overall, our results may serve as novel and sensitive non-invasive biomarkers for renal damage and disease activity. This study adds to our understanding of neutrophil activation and CysC levels in the pathogenesis of SLE. Additionally, the study suggests that targeting CysC and key biomarkers in neutrophil activation networks may play an important role in the treatment of SLE. Future studies with larger sample sizes are required to validate the generalizability of our findings.

### Supplementary Information

**Additional file 1: Data S1.** The differentially expressed proteins between different comparison groups.

**Additional file 2: Figure S1.** ROC analysis indicates that the CysC expression is a biomarker for SLEDAI (A) and kidney involvement (B), with the areas under the ROC curves of 0.672 and 0.729, respectively.

## Data Availability

The mass spectrometry proteomics data have been deposited to the Proteome Xchange Consortium via the PRIDE partner repository with the dataset identifier PXD033144.

## Abbreviations

SLE: Systemic lupus erythematosus
CysC: Cystatin C
SLEDAI: SLE disease activity index
SLE-PN: Patients with proteinuria
SLE-non-PN: Patients without proteinuria
SLE-DP: Patients with anti-dsDNA positive
SLE-non-DP: Patients with anti-dsDNA negative
