# Supplementary material for: Proteomic analyses reveal cystatin c is a promising biomarker for evaluation of systemic lupus erythematosus
Source: Clin Proteomics. 2023 Oct 18;20:43. doi: 10.1186/s12014-023-09434-9 (PMC10583312; doi:10.1186/s12014-023-09434-9)
Supplement: Supplementary file 2 — Additional file 2: Figure S1. ROC analysis indicates that the CysC expression is a biomarker for SLEDAI (A) and kidney involvement (B), with the areas under the ROC curves of 0.672 and 0.729, respectively. [file 12014_2023_9434_MOESM2_ESM.pdf]

A

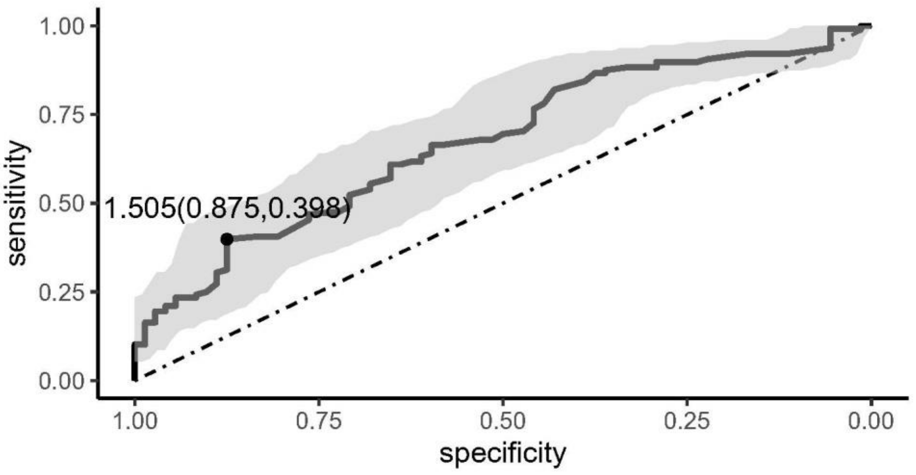

B

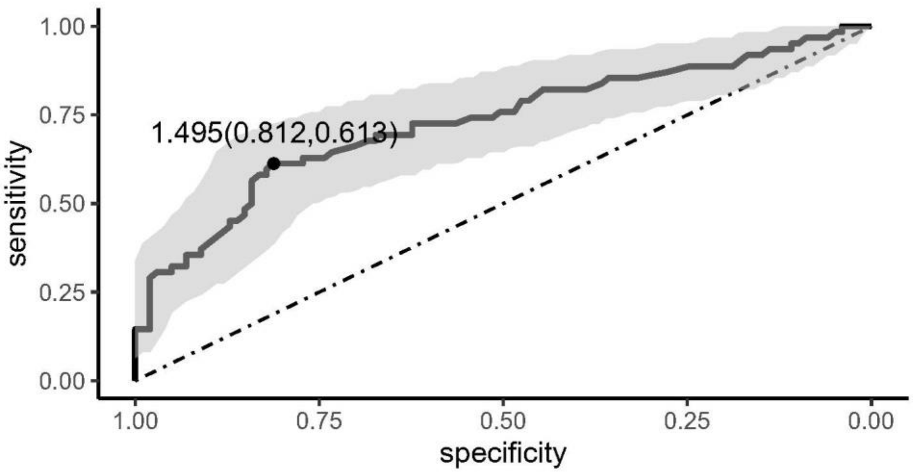

Additional file 2 : Figure S1 CysC serves as a biomarker for disease activity and kidney damage of SLE. ROC analysis indicates that the CysC expression is a biomarker for SLEDAI (A) and kidney involvement (B), with the areas under the ROC curves of 0.672 and 0.729, respectively.
